# Stacking faults and superstructures in a layered brownmillerite

**DOI:** 10.1107/S0108768111042005

**Published:** 2011-11-17

**Authors:** H. Krüger, S. Stöber, T. R. Welberry, R. L. Withers, J. D. Fitz Gerald

**Affiliations:** a Institute of Mineralogy and Petrography, University of Innsbruck, Innsbruck, Austria; b Research School of Chemistry, Australian National University, Canberra, Australia; c Faculty of Geoscience, Martin Luther University of Halle-Wittenberg, Germany; d Research School of Earth Sciences, Australian National University, Canberra, Australia

**Keywords:** layered brownmillerite, diffuse scattering, stacking faults, modulated structure

## Abstract

Single crystals of Ca_4_Fe_2_Mn_0.5_Ti_0.5_O_9_ have been synthesized using a flux method. The structural characterization using single-crystal X-ray diffraction revealed the space group *Amma* and unit-cell dimensions of *a* = 5.3510 (6), *b* = 26.669 (3), *c* = 5.4914 (6) Å. The structure is isotypic with Sr_3_NdFe_3_O_9_ [Barrier *et al.* (2005 ▸). *Chem. Mater. 17*, 6619–6623] and exhibits separated brownmillerite-type layers. One-dimensional diffuse scattering shows that the unit cell is doubled along **c** by alternating the intra-layer order of tetrahedral chains, causing stacking faults along the **b** direction. A computer simulation was performed, proving that the observed intensity variations along the diffuse scattering rods originates from two different local structures depending on the configuration of the tetrahedral chains. Selected-area electron diffraction experiments exhibit well ordered regions characterized by satellite reflections corresponding to two different superstructures. Both superstructures can be described using the superspace group *A*2_1_/*m*(0βγ)0*s*, with γ = 0.5 and β ≃ 0.27 or β = 0.

## Abstract 2

Stacking faults in Ca_4_Fe_2_Mn_0.5_Ti_0.5_O_9_ have been examined using X-ray diffraction and high-resolution transmission electron microscopy. Electron diffraction revealed two superstructures with ordered stacking sequences.

## 1. Introduction   

The ferrite or aluminoferrite phases (Ca
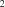
AlFeO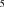) in cements exhibit brownmillerite-type structures. They are important for the hydration process with the ability to react with water (and with additional phases like gypsum), initializing the crystallization of hydration phases like ettringite-type structures (AFt phases) or layered calcium aluminate hydrates (AFm phases). In contrast, perovskite-type structures present in calcium aluminate cements (CAC) remain inert during the hydration process in a hydrous environment. The influence of manganese ore additions to CAC raw materials was investigated by Pöllmann & Oberste-Padtberg (2001 ▸). They demonstrated that certain Mn contents reduce the sintering temperature, lower the costs of the starting materials and extend the early strength. In this context the influence of manganese on the ferrite phase concerning stability and properties of brownmillerite- and perovskite-type phases in the system Ca
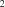
Fe
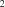
O
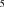
–Ca
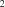
Mn
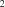
O
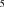
–Ca
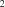
Al
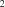
O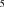 was investigated by Zötzl & Pöllmann (2006 ▸).

Ferrites occur in different types of ordinary Portland cement clinkers and in fused CAC clinkers. In ordinary Portland cement (OPC), brownmillerites coexist with *C* 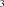 *S*, *C* 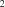 *S* and *C* 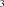 *A* (cement nomenclature: *C* = CaO, *A* = Al
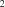
O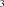, *S* = SiO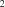, *F* = Fe_2_O_3_) as the main clinker phases and in CAC together with *CA*. The abundance of ferrite phases in OPC and CACs is quite different: OPC clinkers contain 5–15% ferrites, whereas iron-rich CACs may contain up to 20–30% ferrites. Furthermore, the Al/Fe ratio of brownmillerites Ca
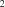
(Fe
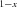
Al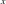)
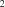
O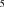 differs significantly: OPCs generally contain Al-rich ferrites in the range *C* 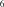 *A* 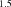 *F* 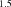
–*C* 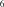 *A* 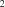 *F*, whereas CACs contain Fe-rich brownmillerites. Brownmillerite phases (ferrites) show important differences compared with pure members of the solid solution series Ca
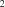
(Fe
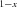
Al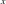)
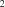
O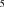 (Fukuda & Ando, 2002 ▸; Redhammer *et al.*, 2004 ▸; Krüger, 2011 ▸), depending on the sintering temperature, the cooling process, the oxygen fugacity (*f*O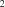) and the chemical composition of the melt. Some of the iron may be reduced to Fe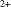 in fused CAC, because of variable *f*O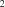 in the kiln (Touzo *et al.*, 2001 ▸). Certain amounts of raw material impurities like MgO, TiO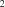, Cr
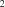
O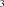, Mn
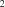
O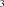, K
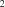
O and Na
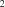
O are incorporated in the brownmillerite crystal structure of the clinker phases. First, a common substitution in ferrite solid solution series can be described as a correlated substitution of trivalent cations Fe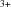 and Al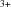 by Mg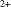 + (Si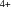, Ti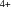) to provide charge balance. Second, trivalent cations are partially substituted by Si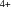 and Ti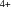 together with an increase of O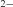 to provide the charge balance (Marinho & Glasser, 1984 ▸). Taylor (1997 ▸) indicated that the chemical compositions of ferrites in OPC are quite constant. He gave a general composition with Ca
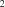
AlFe
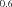
Mg
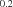
Si
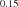
Ti
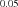
O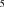 for brownmillerites crystallized in OPC clinkers. Moreover, alumino-ferrite phases show intergrowth with the aluminate phase in OPC (Maki, 1974 ▸). Gloter *et al.* (2000 ▸) investigated the ferrite phase of CAC clinkers by transmission electron microscopy (TEM). The authors found that perovskite lamellae exist in brownmillerite crystals, exhibiting coherent interfaces.

In single-crystal synthesis experiments examining Mn and Ti substitution in brownmillerite we found a phase which has not yet been reported in cements. An isotypic structure has been reported for Sr
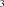
NdFe
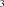
O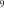 (Barrier *et al.*, 2005 ▸). As these phases [Ca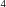 *B* 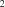 *B*′O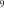 (*B* = Al, Fe; *B*′ = Ti, Mn)] form if a sufficient amount of four-valent species (Mn or Ti) are present, they may occur in cement clinkers. We use the term *layered brownmillerite* because the structure contains separated blocks of the brownmillerite structure. The layered character is evident in the occurrence of stacking faults and the resulting strong one-dimensional diffuse scattering. Furthermore, they share many structural features with brownmillerites, as will be discussed later. The results presented in this study have been derived from one sample of composition Ca
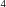
Fe
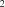
Mn
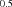
Ti
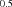
O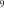. However, we found *layered brownmillerites* (with the same diffuse scattering) in a wide range of the system Ca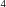 *B* 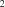 *B*′O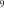 with *B* = Al, Fe; *B*′ = Ti, Mn.

## 2. Experimental   

### 2.1. Synthesis   

Ca
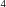
Fe
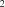
Mn
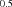
Ti
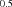
O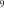 single crystals were synthesized using a flux-growth process (Kahlenberg & Fischer, 2000 ▸; Redhammer *et al.*, 2004 ▸). Pure and pre-dried CaCO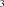 (0.550 g), Fe
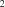
O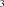 (0.329 g), TiO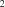 (0.055 g) and MnO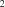 (0.060 g, all compounds Merck 99.9%) were mixed. Pre-dried CaCl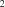 (3 g, Merck 98%) were added and the mixture was homogenized and transferred into a 25 ml Pt-crucible (Pt90Au10). The Pt-crucible was covered with a lid and put into a muffle furnace. The reaction mixture was heated to 1473 K and kept at this temperature level for 3 weeks. Finally, the sample was quenched in ice water. Single crystals were removed from the flux by dissolving it in de-ionized water.

### 2.2. Single-crystal X-ray diffraction   

Suitable crystals of good optical quality were selected from the sample material using a petrographic microscope and subsequently prepared for single-crystal diffraction experiments. The data collection was performed using a two-circle STOE IPDS-2 imaging-plate diffractometer. Orthorhombic symmetry is supported by a merging 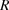 value (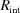) of 0.047 merging in Laue group 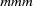 (all reflections, before absorption correction). From the analysis of systematically absent reflections, the extinction symbol 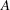--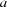 was derived, which is consistent with the space group 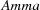 (No. 63) or 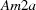 (No. 40; Hahn, 1983 ▸). The non-standard setting was chosen to conform with the most abundant setting used for brownmillerites (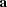 parallel to the tetrahedral chains, 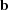 parallel to the stacking direction).

Data reduction including Lorentz–polarization as well as absorption correction (using 10 indexed crystal faces) was undertaken with the software *X-Area* (Stoe & Cie GmbH, 2005*a* ▸) and *X-Red* (Stoe & Cie GmbH, 2005*b* ▸). Details on the data collection and processing are summarized in Table 1 ▸.

Apart from the Bragg reflections a set of one-dimensional diffuse scattering rods are present in the diffraction pattern. These rods are oriented parallel to 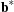 and are completely separated from the Bragg scattering. In reciprocal space sections 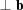 the diffuse streaks show up as sharp satellite reflections, corresponding to doubling the unit cell along **c**.

**Figure 1:**
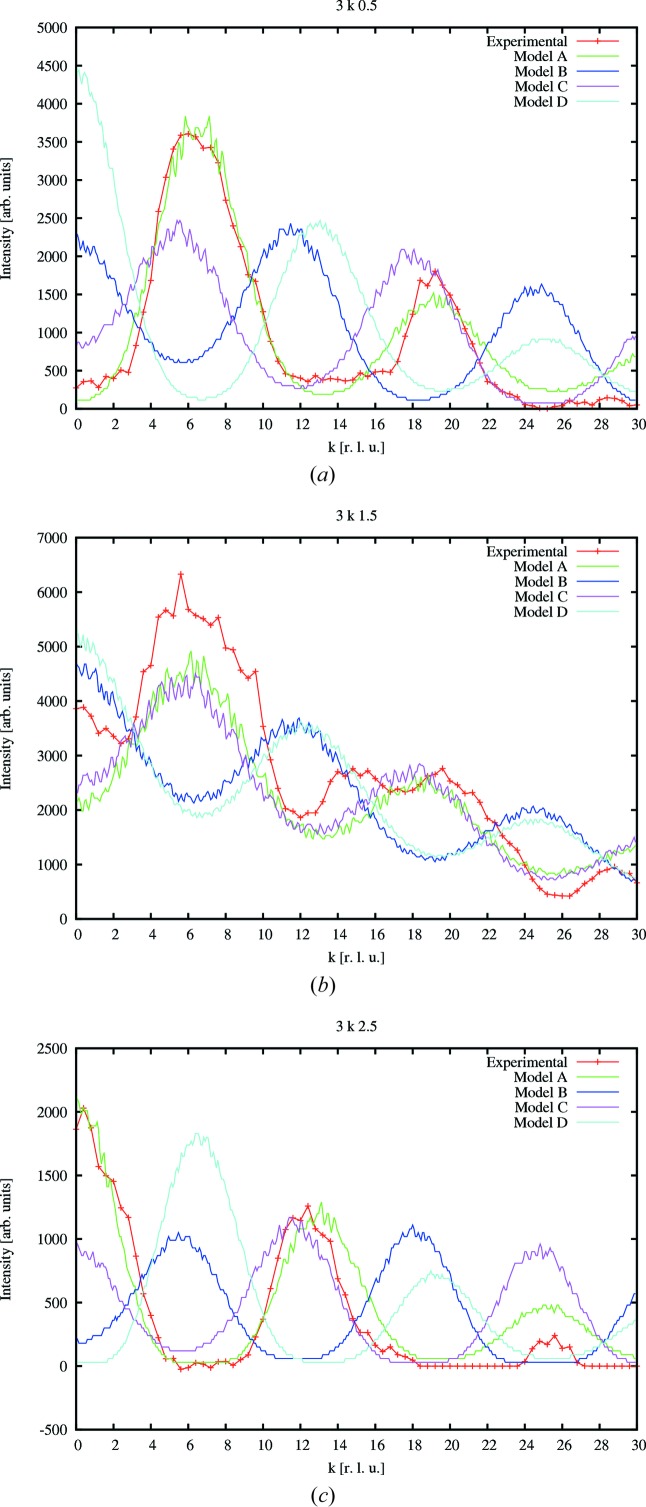
Line profiles of 3*k*0.5, 3*k*1.5 and 3*k*2.5. Experimental diffuse intensity is shown along with line profiles derived from models *A*–*D*.

Another crystal (0.14 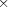 0.09 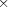 0.06 mm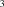) was used for the collection of diffuse scattering data. This data collection was performed using a STOE STADI-4 diffractometer equipped with a point detector (scintillation counter). Monochromated (planar graphite monochromator) Mo *K* 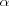 radiation was used, and the sealed tube was powered at 50 kV and 40 mA. The diffractometer was equipped with an 0.5 mm multiple pinhole collimator and the detector-receiving slits were set to 3 mm (= 0.86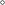, vertical and horizontal). After the orienting matrix was determined, *q*-scans of the diffuse streaks were performed. The streaks 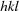 (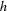 = 1,2,3; 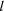 = 0.5, 1.5, 2.5) were measured in the range 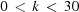, with a stepping of 0.4 reciprocal lattice units. As the maximum counting time was limited to approximately 240 s (due to software limitations; Stoe & Cie GmbH, 1999 ▸), the data of three individual scans (each 240 s counting time per point) was summed up. In order to determine the background, a second set of *q*-scans (for each diffuse streak) was placed parallel and close to the diffuse streak (at the same distance from the origin). These background scans were used to fit background functions of the form 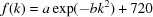, which were subtracted from the corresponding diffuse scattering data. The line profiles with the highest intensities are found in 3*k*0.5, 3*k*1.5 and 3*k*2.5 (Fig. 1 ▸). The measured diffuse scattering data for the remaining lines (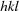, 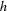 = 1, 2; 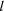 = 0.5, 1.5, 2.5) can be found in the supplementary material.^1^

**Figure 2:**
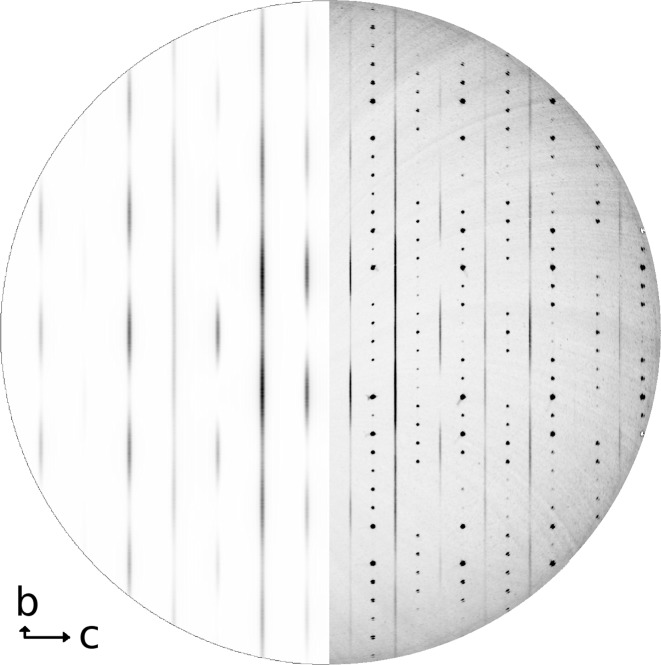
The (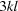) layer: reconstruction from X-ray diffraction data (right, including Bragg reflections). The calculated diffraction pattern (model *A*) is shown on the left; Bragg intensities are subtracted.

As point-detector scans do not reveal the full reciprocal space, an additional experiment on an image-plate diffractometer (MAR345dtb) was employed to measure three-dimensional data. This was done to exclude the effects of twinning or possible overlap of accrued crystal grains, therefore the same crystal was used as for the one-dimensional line scans. The experiment was performed with monochromated Mo *K* 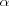 radiation (sealed tube, 45 kV and 40 mA), the beam-size was set by two slit systems to 0.2 
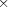
 0.2 mm and the distance between the sample and detector was set to 90 mm. A 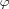-scan of 180° was performed with steps of 0.25° and an exposure time of 1200 s per step. The orienting matrix was determined using *XDS* (Kabsch, 1993 ▸) and the reciprocal space reconstruction was calculated utilizing the software *Xcavate* (Estermann & Steurer, 1998 ▸). Fig. 2 ▸ (right side) shows the experimental data for the (3*kl*) layer, exhibiting the strongest diffuse scattering. The reciprocal space sections (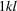), (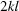), (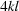) can be found in the supplementary material.

### 2.3. Transmission electron microscopy   

A few grains of the sample material were powdered in an agate mortar and subsequently dispersed on a holey carbon film using ethanol. High-resolution microscopy was performed using a Philips CM300T operated at 300 kV. Images were recorded with a GATAN 1024 
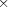
 1024 CCD camera. Further selected-area diffraction experiments were performed using a Philips EM430 operated at 300 kV. Diffraction patterns were recorded on large format sheet film.

## 3. Results   

### 3.1. Average crystal structure   

The average crystal structure was solved in the space group 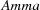 using direct methods (*SIR*97; Altomare *et al.*, 1997 ▸) and subsequently refined with *JANA*2000 (Petříček *et al.*, 2000 ▸). Attempts to solve and refine the structure using the non-centrosymmetric space group 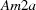 resulted in strong correlation of atomic coordinates of sites which are equivalent in the space group 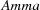. Therefore, the centrosymmetric space group was assumed to be correct.

Four cation sites were identified: two sites with high coordination numbers (8 and 9) which were assigned to Ca atoms. The remaining two sites show tetrahedral and octahedral coordination, consequently these sites were assigned to iron and manganese/titanium, respectively. However, the tetrahedral site is a split position, as is one of its coordinating oxygen sites. The tetrahedra are disordered with respect to two distinct configurations.

Bond-valence sum (BVS; Brown & Altermatt, 1985 ▸) calculations for Fe2 revealed a value of 3.01 (1) and therefore do not show any evidence for four-valent species on the tetrahedral position. Furthermore, refinement with a mixed (Mn,Fe)/Ti occupany showed that no significant amount of Ti is present on the tetrahedral site.

As iron and manganese cannot be distinguished in X-ray diffraction experiments, the sum of iron and manganese was refined as iron. The final refinement included mixed occupancy of Ti/(Fe,Mn) for the octahedral site only, showing that *ca* 25% of that site is occupied by Ti. As the structure requires two three-valent 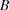 cations per formula unit, it can be assumed that the octahedral site is filled with 50% iron and 25% manganese: giving a formula of Ca
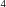
Fe
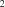
Mn
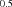
Ti
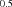
O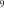. This is in good agreement with the energy-dispersive X-ray spectroscopy (EDX) analysis, which was carried out using an XFLASH 5010 detector (Bruker). As the contrast in scattering factors of (Mn,Fe) and Ti is small and the EDX analysis was not performed quantitatively, the chemical composition should be understood to be only an estimate. Furthermore, mixed-valence states of manganese and iron, as well as non-stoichiometric oxygen content, cannot be excluded. Details of the final refinement can be found in Table 1 ▸, atomic coordinates of the structural model can be found in the supplementary material. Structure drawings were prepared using *DRAWxtl* (Finger *et al.*, 2007 ▸).

The structural model obtained is basically isotypic with the structure of Sr
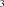
NdFe
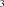
O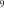 (Barrier *et al.*, 2005 ▸). However, Barrier *et al.* (2005 ▸) decided not to employ a split position for the tetrahedrally coordinated iron, which results in a high distortion of the tetrahedra and a noticeably high bond-valence sum (Brown & Altermatt, 1985 ▸) of 3.18 for Fe2 in Sr
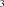
NdFe
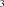
O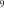 (Barrier *et al.*, 2005 ▸).

**Figure 3:**
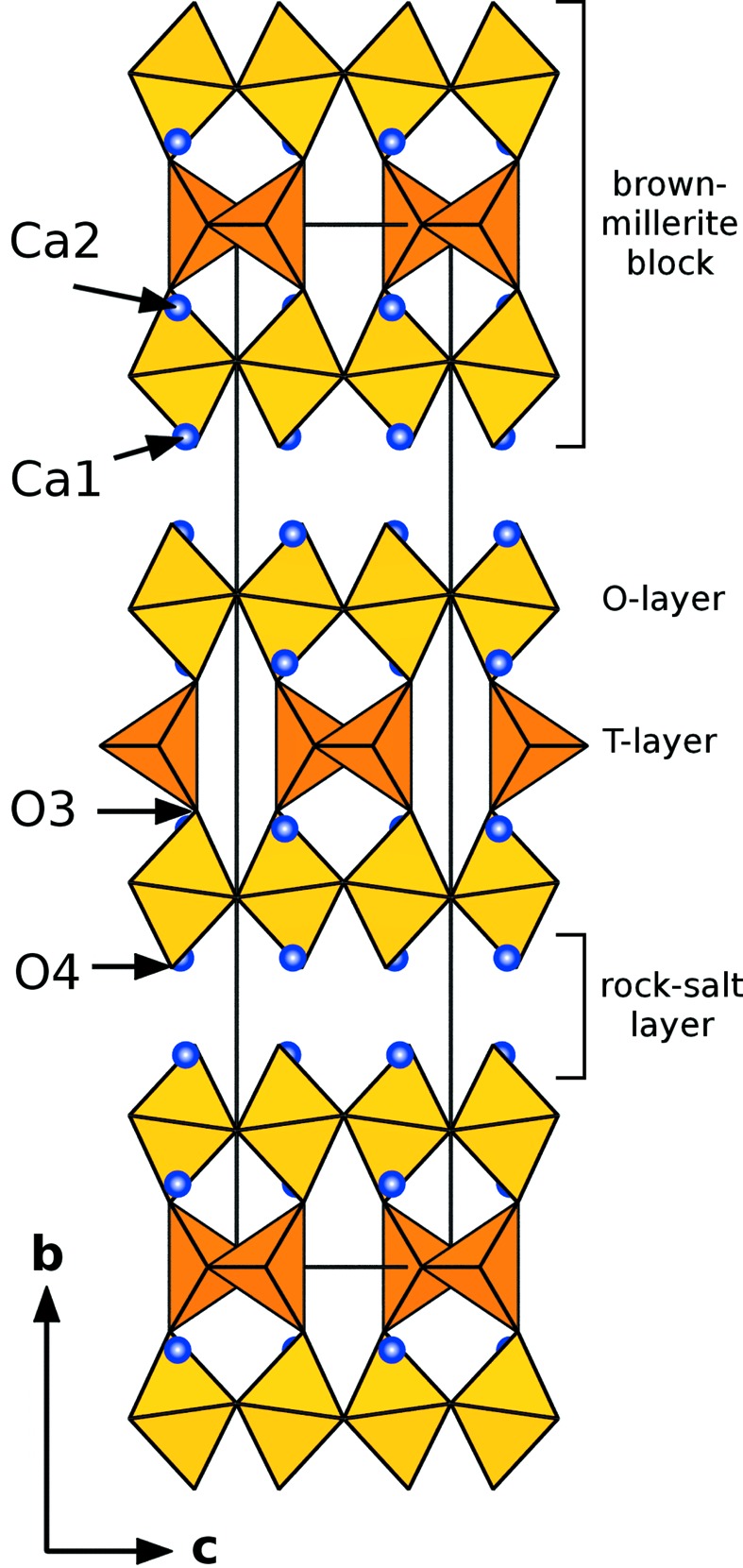
The average crystal structure of Ca
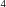
Fe
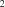
Mn
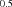
Ti
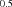
O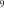. All tetrahedral chains are orientationally disordered and represent two distinct configurations (*cf.* Fig. 2 ▸).

Whereas the brownmillerite structure types exhibit octahedral (*O*) and tetrahedral (*T*) layers connected to form a framework, this structure type shows two-dimensional *O*–*T*–*O* slabs (brownmillerite blocks) separated by distorted rock-salt type CaO layers (see Fig. 3 ▸). Therefore, we use the term *layered brownmillerites* for this structure type. As pointed out by Barrier *et al.* (2005 ▸) this structure type can also be described as an intergrowth between the K
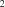
NiF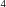 and brownmillerite-type structures.

The coordination of the two Ca atoms show a significant difference: Ca2 is located between the octahedral and the tetrahedral layers, and shows a ninefold coordination and a close to ideal BVS of 2.071 (3). The Ca1 site is within the CaO sheets between the brownmillerite blocks and is slightly under-bonded exhibiting a BVS of 1.845 (4).

### 3.2. Analysis of diffuse X-ray scattering   

**Figure 4:**
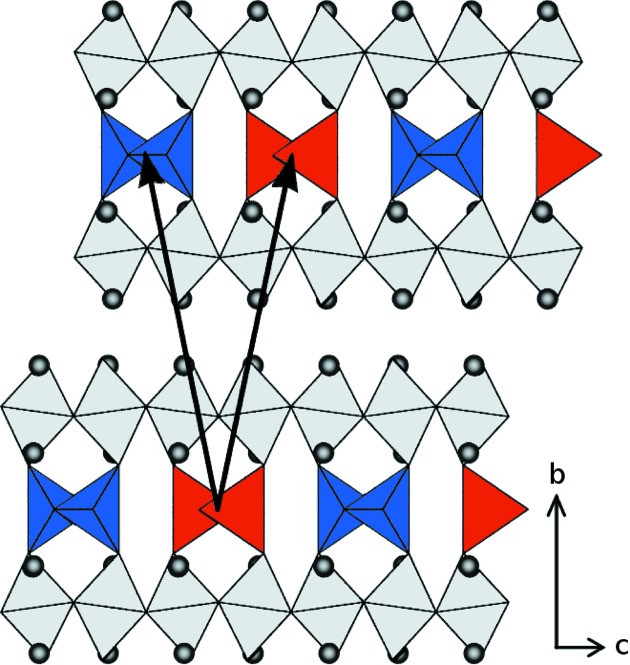
Two neighbouring brownmillerite blocks showing an alternating chain sequence within the tetrahedral layers. Two possible translation vectors are shown: the right one relates the neighbouring layers, the left one would require a shifted position of the upper layer.

The observation of structured one-dimensional diffuse scattering reveals the presence of stacking faults in the structure of the material. However, the average structure shows orientationally disorder of the tetrahedral chains and thus does not allow stacking faults. Therefore, it can be assumed that the chains are not randomly disordered but well ordered. In fact, the type and pattern of the diffuse scattering reveals the ordering scheme of the tetrahedral chains: an alternating sequence of right- and left-handed tetrahedral chains causes the doubling of the 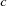 parameter. Therefore, each brownmillerite block may adopt one of two different configurations (Fig. 4 ▸), which allow stacking faults to occur. Two neighbouring tetrahedral layers can be related by two different vectors, namely 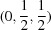 and 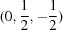, with respect to the cell of the average structure.

The X-ray diffraction data show a smooth continuous intensity distribution (with periodic variations, see Figs. 1 ▸ and 2 ▸) along the lines of diffuse scattering, suggesting random stacking faults, with no preferred stacking sequences.

In order to reveal the structural features which result in the observed intensity variation of the diffuse rods, a computer simulation approach was employed.

The unit cell used to set up the model has a doubled 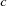 parameter in order to allow an alternating sequence of *R* and *L* chains within the layers. Each unit cell contains two tetrahedral layers, therefore two random variables (0 or 1) are needed (per unit cell) to represent the configuration of the layers.

**Figure 5:**
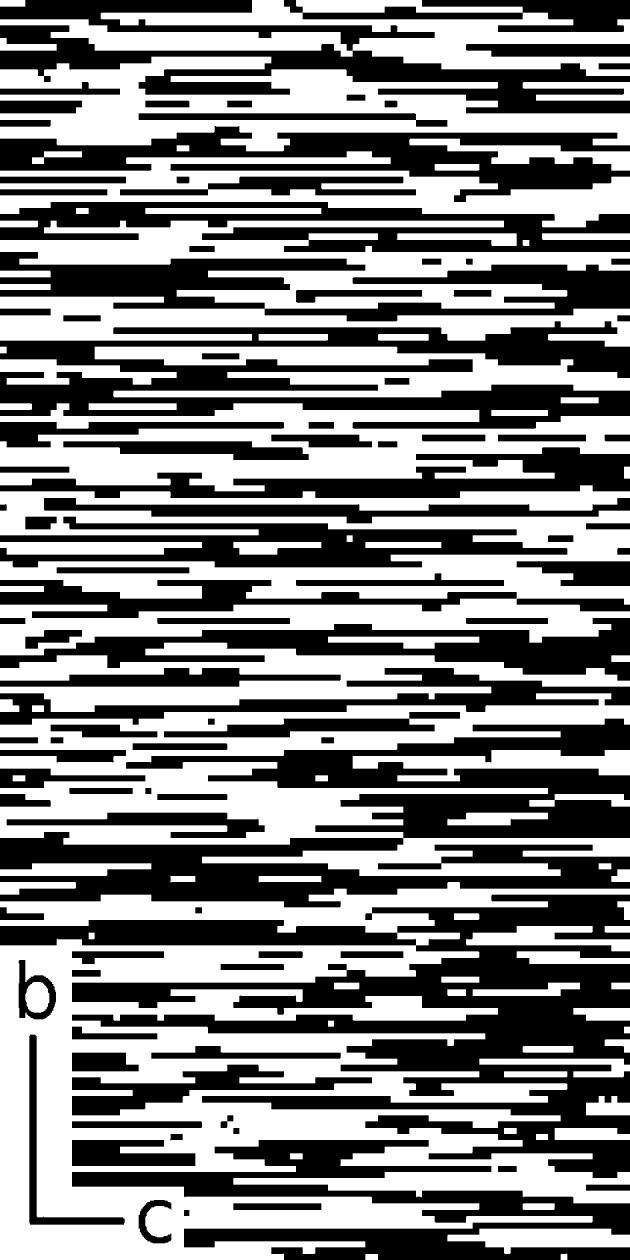
Part (100 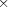 200) of the ‘configuration array’ used to set up the model. Each pixel represents two tetrahedral chains, either *RL* (black) or *LR* (white).

As a system of stacking faults (of perfect two-dimensional layers) represents a strictly one-dimensional disorder problem, very poor statistics are obtained unless a very large one-dimensional sequence can be used. This is not feasible in a relatively small computer model and this inevitably leads to very noisy calculated diffraction patterns. To overcome this problem the system was modelled instead using a two-dimensional disordered array of 512 
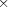
 256 pixels, as shown in Fig. 5 ▸. Each pixel in this figure represents a perfect one-dimensional column of two neighbouring tetrahedral chains along 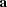. In the (horizontal) 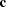 direction a strong nearest-neighbour correlation (of 0.9) is used to induce long rows of like-coloured pixels and this approximates the stacking layers. Black represents the chain configuration *RL* and white represents *LR*. In the vertical 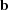 direction the sequence of white and black is random.

The model crystal is built according to this two-dimensional array, using the atomic coordinates of the average structure. However, a few simplifications were made: iron was used for all octahedral and tetrahedral coordinated cations and the displacement parameters were neglected. The positions of atoms represented by split sites in the average structure were chosen in a way as to adopt the configuration required by the configuration array.

The diffraction patterns of the model were calculated utilizing the software *DIFFUSE* (Butler & Welberry, 1992 ▸). The calculation was performed using 1800 lots (randomly chosen subregions of size 1 
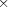
 6 
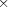
 6 unit cells) assuming periodic boundary conditions. Bragg scattering (average scattering) was determined from 5% of the model crystal and subtracted from the result.

The first model causes continuous rods of diffuse scattering, their positions corresponding to those observed in the experimental data. However, no periodic variation of the intensity along the rods (as present in the experimental data) was produced.

It can be assumed that the local environment of the tetrahedral chains is subject to changes depending on the configuration of the chain, thus leading to two different local environments, which are superimposed in the average crystal structure as derived from X-ray diffraction (XRD) data. Further analysis of the average structure reveals details of the two different configurations: the anisotropic displacement parameters of the Ca atoms, of the bridging (tetrahedra–octahedra) oxygen atoms (O3) and the oxygen atoms opposite (O4) the bridging ones are significantly elongated along **a**. To reveal the magnitude of the displacements these atoms were split (isotropic displacement parameters were used) and another refinement was carried out. The distances between the pairs of split positions are: O3: 0.23, Ca2: 0.15, O4: 0.13 and Ca1: 0.11 Å. The sites closer to the *T* atoms (O3 and Ca2) show the largest displacements, whereas the more distant sites (O4 and Ca1) show smaller separations. Atomic coordinates of the refined split positions are listed in the supplementary material.

To work out the directions of the shifts (*e.g.* which of the two distinct positions of the split atoms corresponds to which chain configuration), considerations on polyhedral distortions were employed, as well as calculated diffraction patterns of the model crystal which were compared with experimentally determined diffuse scattering data. First a definition of the chain configuration/direction is needed. In a projection along **a** (Fig. 4 ▸), the two chain configurations can be easily distinguished: One type of chain has its intra-chain connecting oxygen atoms (O5) pointing along **a** (Fig. 4 ▸, red): these will be referred as type 1 chains; their chain direction is **a**. The type 2 chains have their connecting O atoms pointing in the opposite direction (−**a**, Fig. 4 ▸, blue); the chain direction is −**a**.

**Figure 6:**
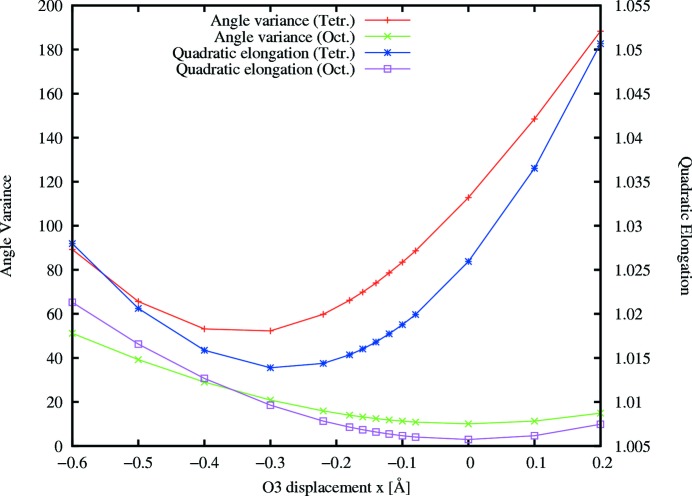
Distortion parameters for displacement of the bridging oxygen O3 (between octahedra and tetrahedra).

As shown by the split-position refinement the bridging oxygen between the tetrahedron and the octahedron (O3) exhibits the largest shift. We calculated distortion parameters (Robinson *et al.*, 1971 ▸) for the tetrahedra and the octahedra depending on the displacement of O3 along the chain direction. Fig. 6 ▸ shows that the tetrahedral distortion has a minimum for a shift slightly below −0.3 Å (negative is against the chain direction). However, the minimal distortion for the octahedra is close to a displacement of 0. Consequently the displacement of O3 is opposite the chain directions, its magnitude being determined by a distortion equilibrium between the octahedra and the tetrahedra.

Given the fact that the direction of the displacement of O3 is well determined by the tetrahedral distortion parameters, we tried to use BVS calculations to reveal the direction of the Ca2 displacement. However, the derived BVS for the two options are not significantly different. Therefore, the displacement of Ca2 has to be determined by analysis of the diffuse scattering.

Four computer models (all combinations of positive and negative displacements for O3 and Ca2, see Table 2 ▸) have been derived and their diffraction patterns were compared with the experimentally derived line profiles. It turns out that all major intensity peaks are well reproduced by model *A* using opposite displacements for O3 and Ca2 (with Ca2 displaced along the chain direction), which shows the best fit to the experimental data. Models *B* and *D* fail to produce the most intense peaks, whereas model *C* does not reproduce the intensities of the peaks and is unlikely because of the tetrahedral distortion parameters. A constant scaling factor is applied to the calculated data as presented in Fig. 1 ▸, which shows the 3
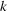
0.5, 3
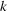
1.5 and 3
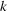
2.5 line profiles as an example. Fig. 2 ▸ compares the experimental and calculated data of the (3*kl*) plane. All other measured line profiles and those derived from the four models are included in the supplementary material.

**Figure 7:**
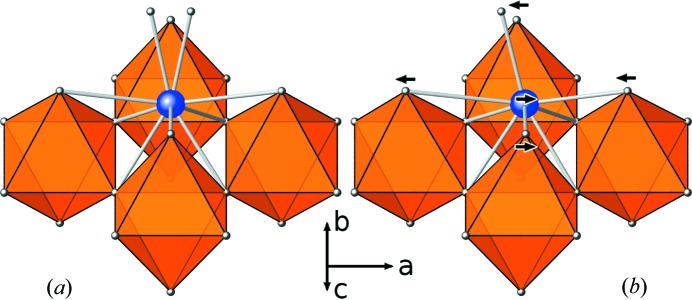
Coordination of the Ca2 site (blue) in Ca
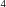
Fe
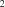
Mn
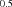
Ti
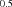
O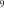. (*a*) Average structure: the split-site O5 can be seen at the top. (*b*) Local structure: only one position of O5 exists, corresponding to one tetrahedral chain configuration. O3 atoms of the same tetrahedral chain are shifted to the left, the O3 belonging to the neighbouring chain, and Ca2 atoms are shifted to the right. This figure is in colour in the electronic version of this paper.

Compared with the average structure the coordination shell of Ca2 is considerably changed. The Ca2 site lies on a mirror plane (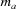) in the average structure, which is reflected by the Ca2—O distances, as shown in Table 3 ▸. However, in the local structure this mirror plane is lost and the coordination polyhedron exhibits stronger distortion. Fig. 7 ▸(*a*) shows the coordination of Ca2 in the average structure, whereas Fig. 7 ▸(*b*) depicts the local environment of Ca2. Black arrows show the direction of the displacements of Ca2, O3 compared with their positions in the average structure. The largest changes are found in the distances to the neighbouring O3 atoms in the 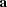 direction. Even though the distances change significantly (see Table 3 ▸), the BVS of Ca2 is just slightly larger.

The displacements of the atoms in the rock-salt layer (O4, Ca1) are smaller and depend not only on one tetrahedral chain, but also on the chain configuration in the next brownmillerite block. Furthermore, a shearing distortion can be assumed due to displacements of O atoms in the equatorial planes of the octahedra. Attempts to model these features of the local structure failed, owing to the limited quality of the data (poor counting statistics, especially in the 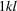 and 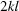 lines with 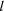 = 0.5, 1.5, 2.5, see supplementary material). Some features in the diffuse lines remain unexplained, *e.g.* maxima at 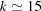 in 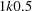, 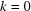 and 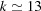 in 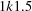, 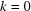 and 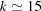 in 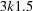. However, the derived model of the local structure explains the dominant intensity variation along the diffuse lines. A future study using synchrotron radiation and an improved data treatment (Lp and absorption correction) might be able to reveal more details of the local structure.

### 3.3. Transmission electron microscopy   

**Figure 8:**
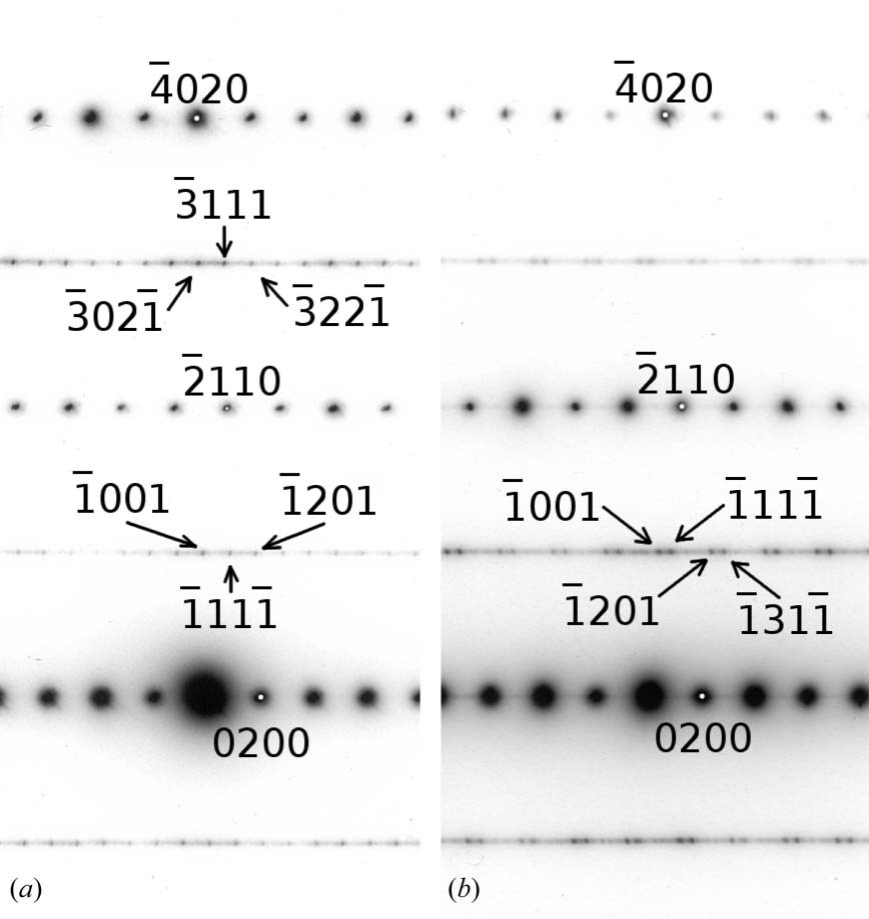
ED patterns of the [102] zone axis, showing two different sets of superstructure reflections. (*a*) Commensurate with 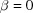 and (*b*) incommensurate with 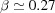. The incommensurate satellites are dominant in the investigated sample.

In contrast to the XRD results, selected-area electron diffraction revealed that the samples show additional sharp peaks located on the diffuse streaks, which indicates a certain degree of long-range order with respect to the corresponding stacking sequences (see Fig. 8 ▸).

Two different patterns of superstructure reflections were observed along the [102] zone axis, as shown in Fig. 8 ▸. The electron-diffraction patterns were recorded from different regions of the same grain. Both show the same pattern of main reflections and diffuse rods parallel to 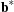. However, satellite reflections located on the diffuse streaks show different positions. For the pattern shown in Fig. 8 ▸(*b*) a **q** vector^2^ of 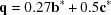 can be used to index the superstructure reflections. In Fig. 8 ▸(*a*) a commensurate **q** vector of 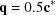 is sufficient to index the pattern. However, the incommensurate **q** vector seems to be dominant in the investigated sample.

All observed electron diffraction (ED) patterns show a 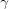 component of 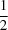, resulting in an alternating intra-layer chain sequence. However, two different values for 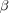 were observed, 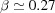 and 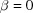, each of them corresponding to a certain stacking sequence.

The monoclinic stacking sequences allow for twinning according to a mirror plane 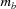, which was frequently observed. In Fig. 8 ▸(*b*) very weak satellites can be seen which cannot be indexed with 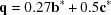, but with a mirror-related vector 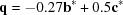.

**Figure 9:**
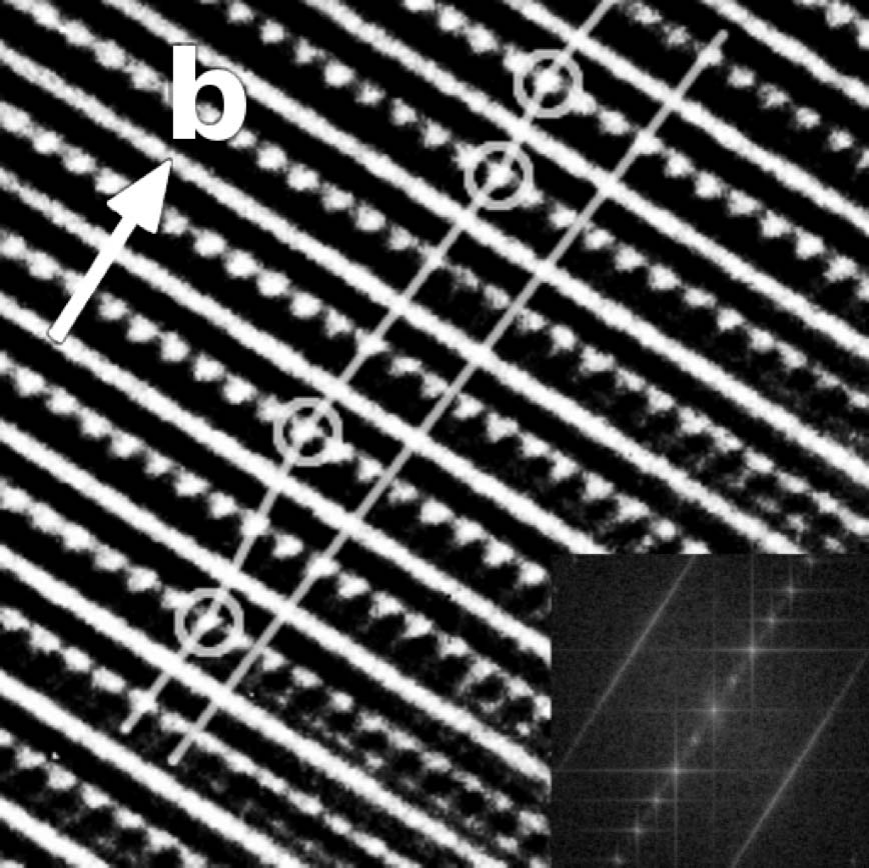
HREM image recorded along the [102] zone axis. The length of the arrow corresponds to 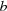. The Fourier transform is derived from a larger area [*ImageJ* (Abràmoff *et al.*, 2004 ▸) was used for image processing].

As demonstrated by other authors (Krekels *et al.*, 1993 ▸; D’Hondt *et al.*, 2008 ▸) the [102] zone axis represents a special direction, which gives (for geometrical reasons; see Fig. 17 of Krekels *et al.*, 1993 ▸, for details) a strong contrast for different tetrahedral chain configurations. The same is true for the *layered brownmillerite* structure, since the same geometry applies for the brownmillerite blocks. Consequently, high-resolution electron microscopy (HREM) images were recorded along the [102] zone axis (Fig. 9 ▸). The image shows smooth lines perpendicular to 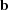, which are likely to be caused by the rock-salt layers. The distance between these corresponds to 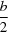. In between these lines the dotted contrast represents the alternating tetrahedral chains. Their spacing (dot-to-dot) is *ca* 4.8 Å, which agrees with the doubled 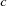 lattice in this projection. The straight line is a projection of 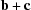 (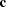 as in the average structure), which intersects a distinct tetrahedral chain at each tetrahedral layer. As can be seen along the line, it intersects either bright or dark spots in an irregular sequence. The staggered line follows the stacking vector layer by layer and every change in the stacking vector is marked with a circle. By correlating five HREM images, we were able to map the stacking vectors between 45 brownmillerite blocks: − − + − + + + + + − − − + + − + + + + − + + + − − + − − − + + − − + + + − + + − + − + + (where + and − denote the two possible stacking vectors). As shown later, this sequence is not compatible with any of the observed superstructures and thus represents a region with stacking faults.

### 3.4. Superspace model   

As the observed electron-diffraction patterns can be indexed using different **q** vectors, it is possible to describe the structures utilizing the (3 + 1)-dimensional superspace approach (van Smaalen, 2007 ▸; Janssen *et al.*, 2004 ▸). In order to find a unified superspace model for the different superstructures a monoclinic subgroup of 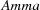 has to be considered as the basic three-dimensional space group. The direction of the unique monoclinic axis has to be parallel to the chain direction. Furthermore, the (3 + 1)-dimensional space group should exhibit an internal phase shift of 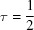 connected with the mirror plane, the effect of this will be described later. The superspace group 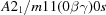 (which is a non-standard setting of No. 11.2; Janssen *et al.*, 2004 ▸) exhibits the desired properties. The disorder of the tetrahedral chains, as implied by the 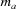 mirror plane, is now solved by the fact that the two mirror-related chains (*R* and *L*) are subject to an internal phase shift 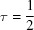. The two atoms (Fe2, O5) which exhibit split positions due to the 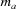 mirror plane are modelled with crenel-type occupational modulation functions (Petříček *et al.*, 1995 ▸, 2000 ▸) of width 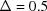. The two symmetry-equivalent positions of the two mentioned atoms are phase-shifted by 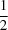. To ensure that they cannot occur in any real-space section at the same time the centres of the crenel functions (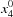) have to be carefully chosen: let us consider two consecutive *T* atoms in the tetrahedral chain, that is for example Fe2 and its symmetry-equivalent position generated by the 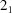-screw axis along 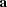. The Fe2 atom takes the position (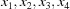), whereas that generated by the screw axis is located at (
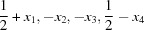
) (note the effect of the superspace symmetry on 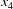). These two atoms shall coexist in every real-space section, therefore the phases of their modulation functions need to be the same. As the phase 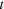 is given by 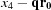 (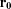 is the position vector of the atom in the basic structure), the phase of the first atom is 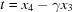. The phase of the next *T* atom along the chain is 
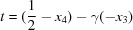
. Putting these into an equation and solving it for 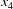


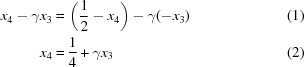
yields the values to be used as the 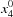 parameters of the crenel functions. The values of 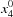 (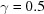) are: 0.34135 and 0.3174 for Fe2 and O5, respectively.

A possible stacking sequence resulting from this structural model (for 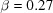) is − + + + + − + + + − + + + − + + + + − + + + − + + + − (where + and − denote the two possible stacking vectors). The sequence exhibits groups of 3 or 4 identical vectors interrupted by single vectors of the other direction. For 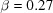 *ca* 35% of the groups have four identical vectors. If this is compared with the sequence obtained by HREM, it can be noted that the observed sequence also shows groups of two or five identical stacking vectors, which cannot occur in an incommensurate sequence if 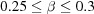. Consequently, the observed sequence includes stacking faults as supported by the Fourier transform of the HREM images, which show diffuse streaks. However, as evident by the ED images showing sharp satellite reflections, long-range ordered regions exist.

## 4. Discussion   

**Figure 10:**
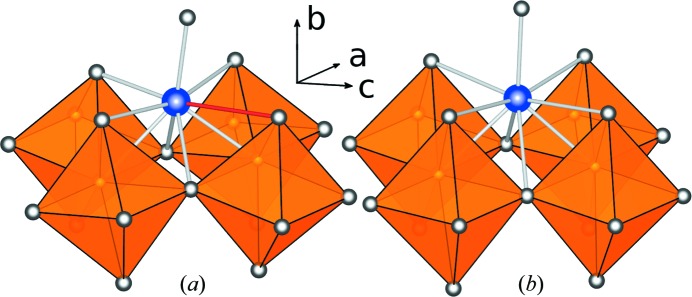
Coordination of the 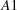 site in (*a*) Ca
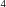
Fe
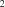
Mn
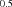
Ti
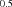
O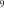 and (*b*) Sr
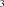
NdFe
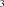
O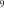.

Comparing the structure of Ca
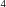
Fe
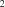
Mn
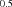
Ti
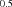
O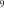 with Sr
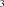
NdFe
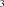
O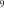 highlights a few significant differences. Whereas the 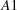 cation site in Sr
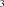
NdFe
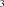
O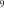 (Sr/Nd site in the rock-salt layer) shows a ninefold coordination, as can be expected for this site, the corresponding Ca site in Ca
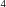
Fe
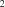
Mn
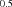
Ti
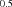
O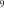 shows an eightfold coordination. A closer look reveals that it is in fact an 8 + 1 coordination with one unusual long bond. For a list of distances see Table 4 ▸. Fig. 10 ▸ compares the 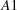 sites in Ca
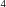
Fe
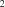
Mn
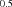
Ti
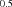
O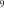 (Fig. 10 ▸ *a*) and Sr
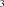
NdFe
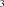
O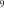 (Fig. 10 ▸ *b*). In Ca
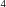
Fe
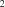
Mn
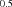
Ti
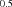
O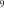 the octahedra show a larger rotation around the 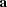 axis, which obviously distorts the coordination shell of the 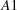 site. The bond shown in red is expanded up to 3.112 (2) Å. The angle between two neighbouring octahedra (measured as the O1—O2—O1 angle along 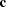) is 163.9°, which is much closer to brownmillerite compounds such as Ca
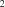
(Fe
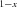
Al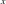)
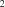
O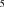 than to Sr
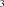
NdFe
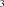
O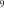, which shows a higher value of 175.5° (Barrier *et al.*, 2005 ▸). This angle changes with the size of the 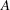 cations as can be seen in brownmillerites: 160.4° is found in Ca
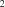
Fe
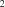
O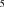 (Redhammer *et al.*, 2004 ▸), whereas in Sr
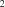
Fe
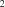
O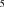 this angle exhibits a value of 170.3° (Berastegui *et al.*, 1999 ▸).

Another striking difference between Ca
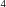
Fe
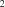
Mn
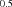
Ti
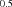
O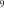 and Sr
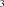
NdFe
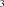
O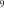 is the octahedral distortion along the stacking direction. In Sr
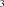
NdFe
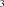
O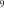 the O3—O4 distance is 4.5 Å compared with 4.08 Å in Ca
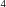
Fe
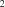
Mn
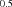
Ti
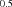
O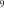. Furthermore, in Sr
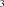
NdFe
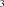
O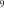 the octahedral site is moved significantly towards the rock salt layer (Fe1—O4 = 2.51 and Fe1—O3 = 1.99 Å). Consequently, the coordination was described as 5 + 1 by Barrier *et al.* (2005 ▸). In Ca
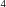
Fe
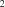
Mn
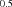
Ti
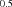
O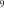 this asymmetry is less pronounced (Fe1—O3 = 2.17 and Fe1—O4 = 1.9 Å).

As there are two different species of 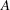 cations in Sr
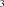
NdFe
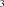
O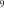 (Sr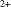 and Nd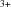) one could expect partitioning of these on the two available sites. However, neutron scattering as used by Barrier *et al.* (2005 ▸) is not able to resolve this issue. BVS calculations do not reveal any significant site preference of Sr or Nd.

Phases of the Ruddlesden–Popper (RP) family (Ruddlesden & Popper, 1958 ▸; Beznosikov & Aleksandrov, 2000 ▸) may be used for comparison, since they exhibit similar rock-salt layers. In the 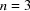 RP phase (Ca
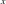
Sr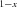)
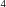
Ti
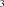
O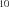 with Ca/Sr as 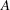 cations, a preference of Sr for the 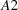 site within the perovskite block is observed (Hawkins & White, 1991 ▸). However, the 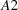 site in 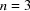 RP phases is smaller than the 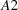 site within the brownmillerite blocks.

In contrast to brownmillerites and the layered brownmillerites, known 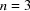 RP phases (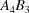
O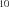 with 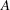 = Ca, Sr and 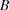 = Mn, Ti; Battle *et al.*, 1998 ▸; Fawcett *et al.*, 1998 ▸; Hawkins & White, 1991 ▸; Elcombe *et al.*, 1991 ▸) show a significant rotation of the octahedra around the 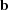 axis (stacking direction).

The general superspace model presented for the *layered brownmillerites* is closely related to the superspace model derived for Sr
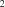
Fe
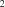
O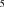 by D’Hondt *et al.* (2008 ▸). The modulation wavevector in both models describes the same relation between intra-layer chain order and the stacking sequence of the layers. In the structure presented here the distance between adjacent tetrahedral layers is almost twice as large as in Sr
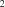
Fe
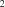
O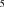 (7.8 Å in Sr
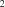
Fe
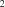
O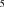, 13.3 Å in the structure presented here). In this context it is interesting to note, nevertheless, that interaction between tetrahedral layers is strong enough to form a certain amount of long-range order.

The displacements derived for O3 and Ca2 by split-atom refinement and analysis of diffuse scattering are in agreement with displacements found in the modulated high-temperature structures of the brownmillerites Ca
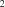
(Fe
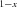
Al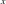)
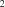
O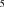 (Krüger & Kahlenberg, 2005 ▸; Lazic *et al.*, 2008 ▸; Krüger, 2011 ▸). In these structures a modulated intralayer sequence of *R* and *L* chains exists and the displacements of the neighbouring atoms (corresponding to O3 and Ca2; in Ca
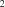
Fe
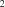
O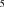 it is O2 and Ca1; Krüger & Kahlenberg, 2005 ▸) can be derived from the amplitudes of their modulation functions (in 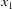): 0.21 and 0.16 Å for O2 and Ca1. Furthermore, the modulation functions are in an anti-phase relation (see Fig. 7 ▸ of Krüger & Kahlenberg, 2005 ▸), which agrees with the result that O3 and Ca2 are displaced in opposite directions.

## 5. Conclusion   

An interesting aspect of this study is the differing results obtained by XRD and ED. X-ray diffraction on the single-crystal scale does not show evidence for any order in the stacking sequence. The XRD pattern can be fully explained by random stacking faults. However, selected-area electron diffraction reveals a high degree of long-range order: regions with order corresponding to at least two different superstructures were observed, although all ED images also show diffuse lines resulting from stacking faults. Furthermore, HREM images prove the existence of fairly disordered regions. All of the above can be understood assuming that *layered brownmillerites* exhibit a structural inhomogeneity, where ordered and disordered regions of limited size co-exist. Electron diffraction is able to detect these ordered regions, because of the stronger interaction between electrons and the sample. Furthermore the electron beam probes the sample on a much smaller volume compared with the XRD experiments. Similar observations have been reported by Schmitt *et al.* (2010 ▸).

The *layered brownmillerites* prove that the interactions between the tetrahedral layers are still strong enough to form order, even if the distance between the *T* layers is about twice as large as in brownmillerites.

## Supplementary Material

Crystal structure: contains datablock(s) I. DOI: 10.1107/S0108768111042005/sn5108sup1.cif

Structure factors: contains datablock(s) I. DOI: 10.1107/S0108768111042005/sn5108Isup2.hkl

Extra figures and tables. DOI: 10.1107/S0108768111042005/sn5108sup3.pdf

## Figures and Tables

**Table 1:** Experimental details

Crystal data	
Chemical formula	Ca_4_Fe_2_Mn_0.5_Ti_0.5_O_9_
*M*_r_	467.40
Crystal system, space group	Orthorhombic, *Amma*
Temperature (K)	298
*a*, *b*, *c* ()	5.3510 (6), 26.669 (3), 5.4914 (6)
*V* (^3^)	783.64 (15)
*Z*	4
Radiation type	Mo *K*
(mm^1^)	7.58
Crystal size (mm)	0.09 0.07 0.05
	
Data collection	
Diffractometer	Stoe IPDS 2
Absorption correction	Integration Stoe *X-RED*32 1.31
*T*_min_, *T*_max_	0.529, 0.643
No. of measured, independent and observed [*I* > 3(*I*)] reflections	3489, 571, 543
*R*_int_	0.029
	
Refinement	
*R*[*F* ^2^ > 2(*F* ^2^)], *wR*(*F* ^2^), *S*	0.023, 0.034, 2.61
No. of reflections	571
No. of parameters	54
No. of restraints	0
_max_, _min_ (e ^3^)	0.48, 0.39

Computer programs used: Stoe *X-AREA*, Stoe *X-RED*, *SIR*97 (Altomare *et al.*, 1997 ▸), *JANA*2000 (Petek *et al.*, 2000 ▸), *DRAWxtl* (Finger *et al.*, 2007 ▸).

**Table 2:** Displacements (in ) for models *A* *D* Positive: in chain direction; negative: opposite to chain direction.

Model	O3	Ca2
*A*	0.11	0.08
*B*	0.11	0.08
*C*	0.11	0.08
*D*	0.11	0.08

**Table 3:** Ca2O distances () in the average and local structure

	Average	Local
Ca2O1	2.4388 (13)	2.485
Ca2O1^i^	2.4388 (13)	2.397
Ca2O2	2.5288 (16)	2.572
Ca2O2^i^	2.5288 (16)	2.487
Ca2O3^ii^	2.7242 (5)	2.537
Ca2O3	2.7242 (5)	2.911
Ca2O3^iii^	2.315 (2)	2.315
Ca2O5	2.3108 (16)	2.326
Ca2O5^i^	2.3108 (16)	
Ca2	2.071 (3)	2.091

Symmetry codes: (i) 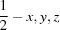; (ii) 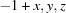; (iii) 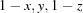.

**Table 4:** Bond distances () Note that the Fe1 site also contains Mn and Ti.

Fe1O1^i^	1.9235 (4)	Ca1O1	2.6901 (17)
Fe1O1^ii^	1.9235 (4)	Ca1O1^ii^	2.6901 (17)
Fe1O2^i^	1.9364 (3)	Ca1O2	2.5004 (14)
Fe1O2^ii^	1.9364 (3)	Ca1O2^ii^	2.5004 (14)
Fe1O3	2.175 (3)	Ca1O4^iii^	2.6969 (3)
Fe1O4	1.903 (3)	Ca1O4	2.6969 (3)
Fe2O3	1.821 (2)	Ca1O4^iv^	2.256 (3)
Fe2O3^v^	1.821 (2)	Ca1O4^vi^	2.404 (2)
Fe2O5	1.918 (4)	Ca1O4^vii^	3.112 (2)
Fe2O5^viii^	1.909 (4)		

Symmetry codes: (i) 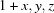; (ii) 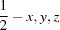; (iii) 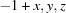; (iv) 
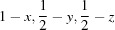
; (v) 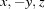; (vi) 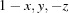; (vii) 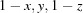; (viii) 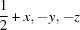.

## Footnotes

1 Supplementary data for this paper are available from the IUCr electronic archives (Reference: SN5108). Services for accessing these data are described at the back of the journal.

2
 (Janssen *et al.*, 2004 ▸).
