# Supplementary material for: Stacking faults and superstructures in a layered brownmillerite
Source: Acta Crystallogr B. 2011 Nov 17;67(Pt 6):476–85. doi: 10.1107/S0108768111042005 (PMC3222140; doi:10.1107/S0108768111042005)
Supplement: Supplementary file 3 [file b-67-00476-sup3.pdf]

Stacking faults and superstructures in a layered brownmillerite

Supplementary Material

H. Krüger[1,2,*], S. Stöber[3], T. R. Welberry[2], R. L. Withers[2], and J. D. Fitz Gerald[4]

[1]*Institute of Mineralogy und Petrography, University of Innsbruck, Austria*
[2]*Research School of Chemistry, The Australian National University, Canberra*
[3]*Faculty of Geoscience, Martin Luther University of Halle-Wittenberg, Germany*
[4]*Research School of Earth Sciences, The Australian National University, Canberra*

October 10, 2011

Abstract

Single crystals of $Ca_4Fe_2Mn_{0.5}Ti_{0.5}O_9$ have been synthesised using a flux method. The structural characterisation using single-crystal X-ray diffraction revealed space group *Amma* and unit cell dimensions of $a = 5.3510(6)$, $b = 26.669(3)$, $c = 5.4914(6)$Å. The structure is isotypic with $Sr_3NdFe_3O_9$ as reported by Barrier *et al.* (*Chem. Mater.* 2005, **17**, 6619) and exhibits separated brownmillerite-type layers. One-dimensional diffuse scattering shows that the unit-cell is doubled along c by alternating intra-layer order of tetrahedral chains, causing stacking faults along the b direction. A computer simulation was performed, proving that the observed intensity variations along the diffuse scattering rods originates from two different local structures depending on the configuration of the tetrahedral chains. Selected area electron diffraction experiments exhibit well-ordered regions characterised by satellite reflections corresponding to two different superstructures. Both superstructures can be described using the superspace group $A2_1/m(0\beta\gamma)0s$, with $\gamma = 0.5$ and $\beta \approx 0.27$ or $\beta = 0$.

*Corresponding author. Email: Hannes.Krueger@uibk.ac.at

Table 1: Atomic coordinates, U_{eq} and x_4^0

Atom	sof	x	y	z	U_{eq}	x_4^0 for $\gamma = 0.5$
Fe1 (Fe,Mn)	0.749(14)	0.75	0.142345(16)	0.24550(6)	0.01173(16)	
Ti1	0.251(14)	0.75	0.142345(16)	0.24550(6)	0.01173(16)	
Fe2	0.5	0.69589(11)	0	0.18271(13)	0.0131(2)	0.34135
Ca1	1	0.25	0.20345(3)	0.23793(10)	0.01771(19)	
Ca2	1	0.25	0.07962(2)	0.27533(11)	0.01629(18)	
O1	1	0	0.13093(8)	0	0.0164(5)	
O2	1	0	0.14546(8)	0.5	0.0159(5)	
O3	1	0.75	0.06194(9)	0.3119(4)	0.0221(6)	
O4	1	0.75	0.21301(9)	0.1973(4)	0.0202(6)	
O5	0.5	0.3409(7)	0	0.1349(7)	0.0150(10)	0.3174
split refinement						
Ca1	0.5	0.2602(7)	0.20347(3)	0.23798(11)	0.0169(2)	
Ca2	0.5	0.2642(5)	0.07961(3)	0.27530(11)	0.0142(2)	
O3	0.5	0.7720(10)	0.06190(10)	0.3118(4)	0.0172(6)	
O4	0.5	0.762(2)	0.21293(10)	0.1971(4)	0.0189(7)	

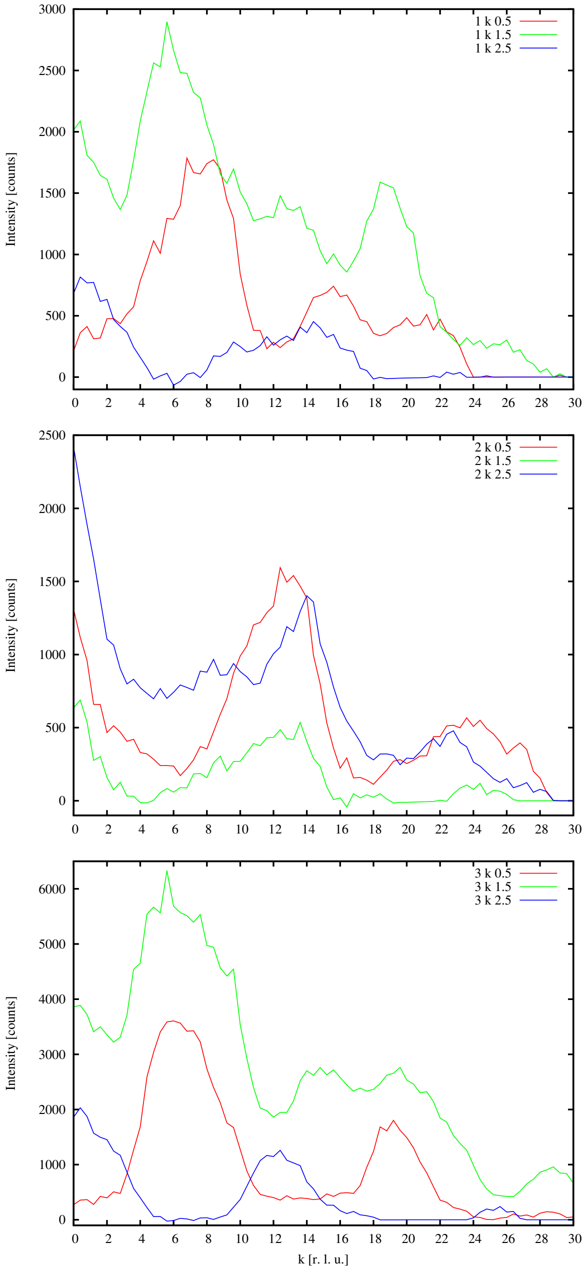

Figure 1: Intensity distribution in the rods of diffuse scattering. Background-corrected data from point detector q-scans along b^* performed on a Stoe Stadi-4 diffractometer (50kV, 40mA). Data has been collected with a spacing of 0.4 r. l. u. and an integration time of 720 sec.

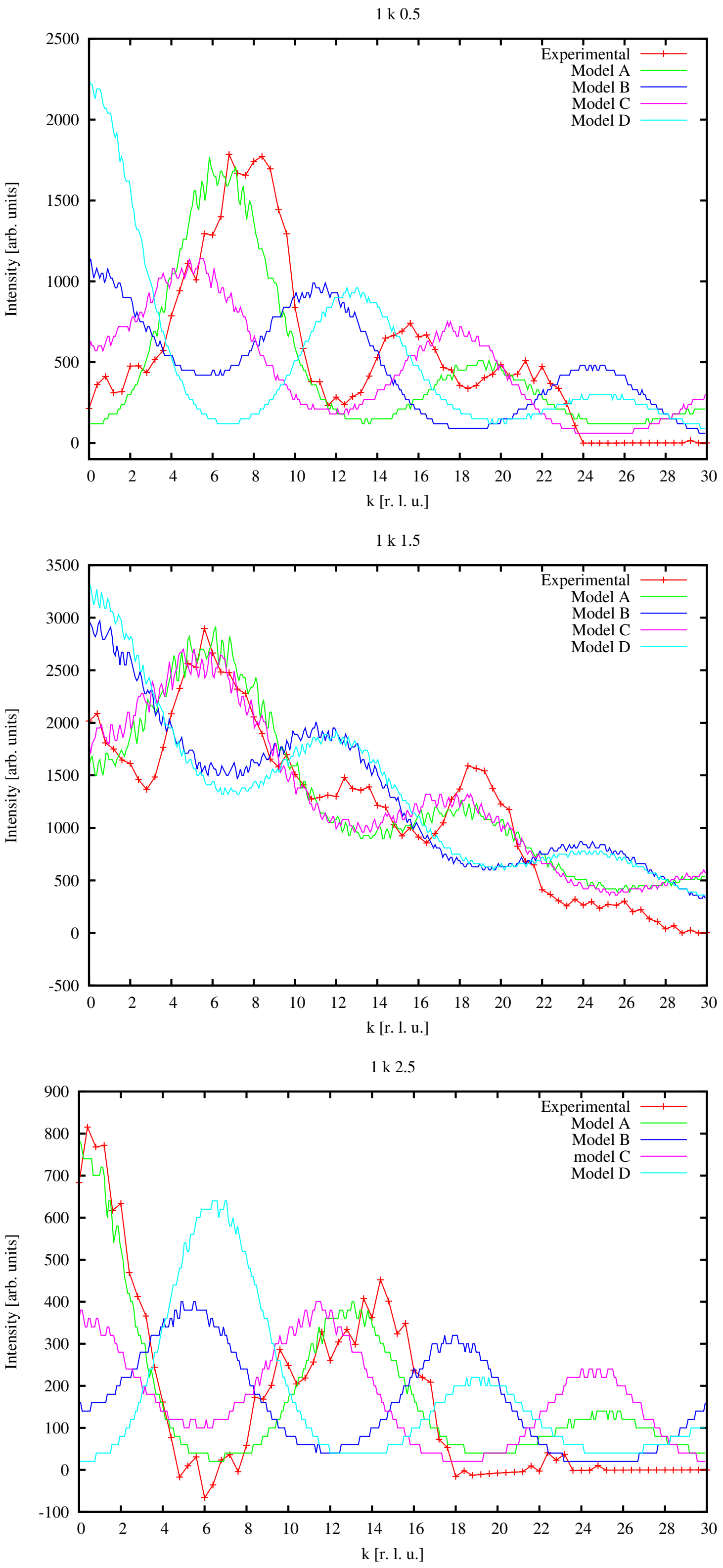

Figure 2: Line profiles of 1k0.5, 1k1.5 and 1k2.5. Experimental diffuse intensity is shown along with line profiles derived from models A–D.

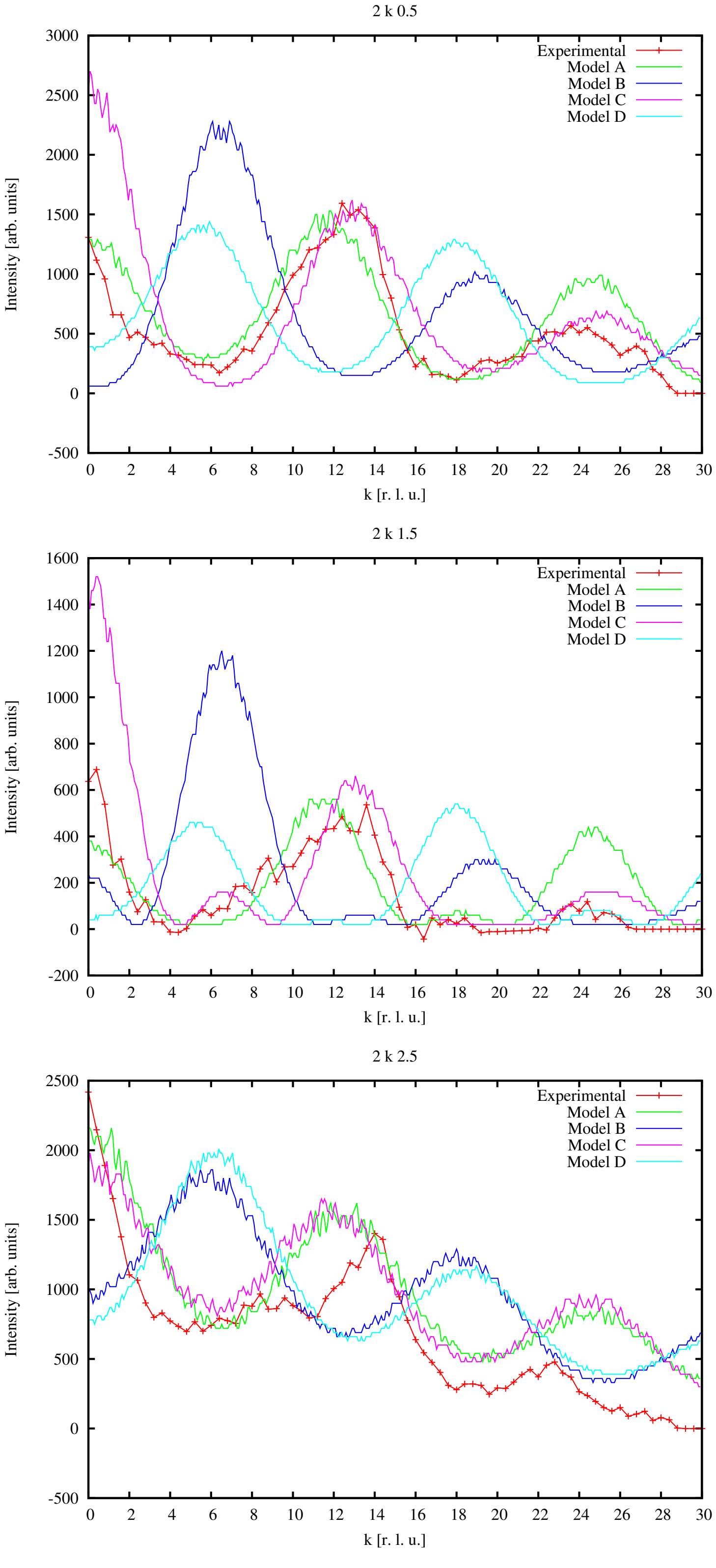

Figure 3: Line profiles of 2k0.5, 2k1.5 and 2k2.5. Experimental diffuse intensity is shown along with line profiles derived from models A–D.

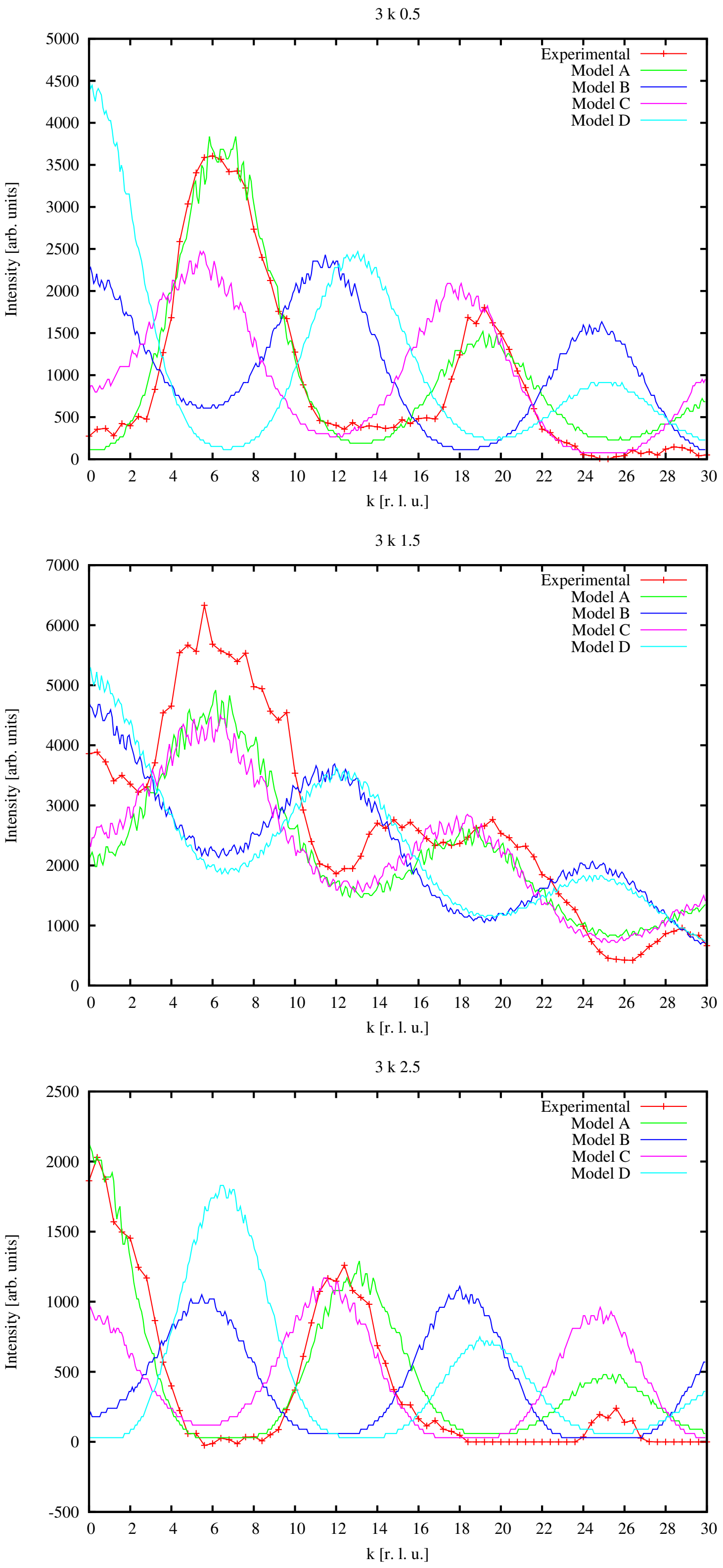

Figure 4: Line profiles of 3k0.5, 3k1.5 and 3k2.5. Experimental diffuse intensity is shown along with line profiles derived from models A–D.

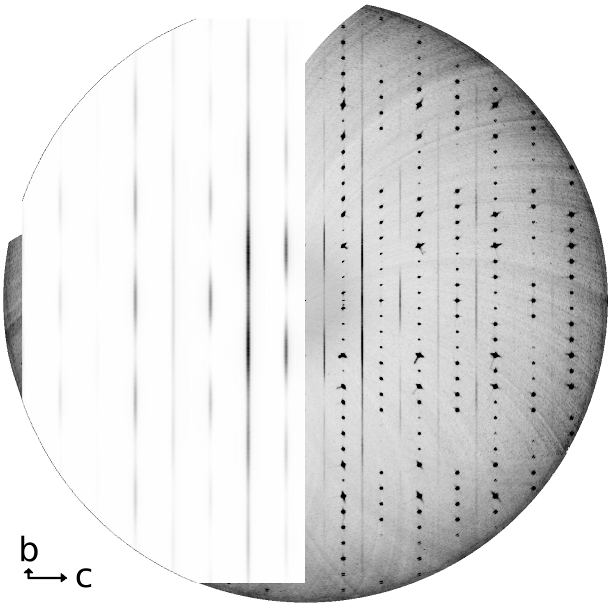

Figure 5: The $(1kl)$ layer: reconstruction from X-ray diffraction data (right, including Bragg reflections). The calculated diffraction pattern (model A) is shown on the left; Bragg intensities are subtracted.

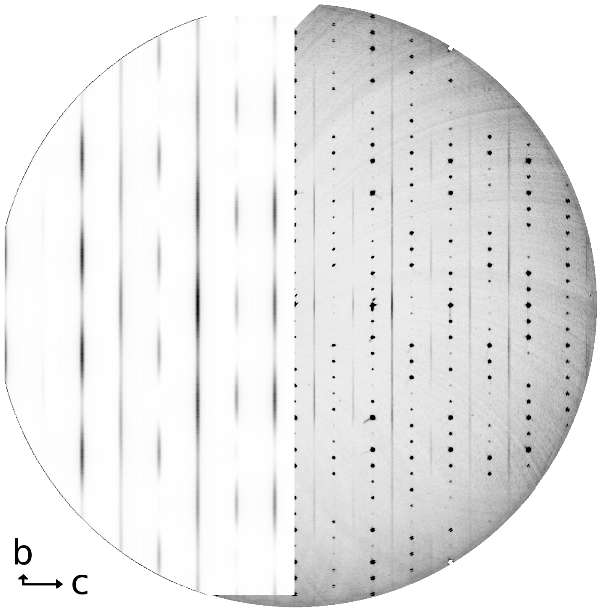

Figure 6: The $(2kl)$ layer: reconstruction from X-ray diffraction data (right, including Bragg reflections). The calculated diffraction pattern (model A) is shown on the left; Bragg intensities are subtracted.

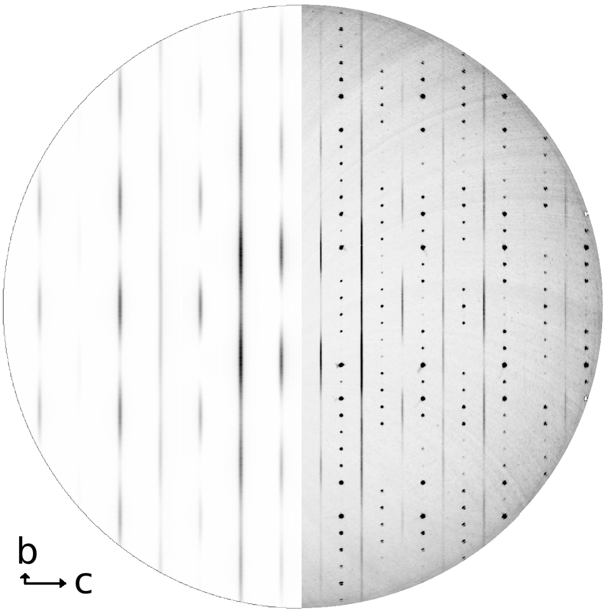

Figure 7: The $(3kl)$ layer: reconstruction from X-ray diffraction data (right, including Bragg reflections). The calculated diffraction pattern (model A) is shown on the left; Bragg intensities are subtracted.

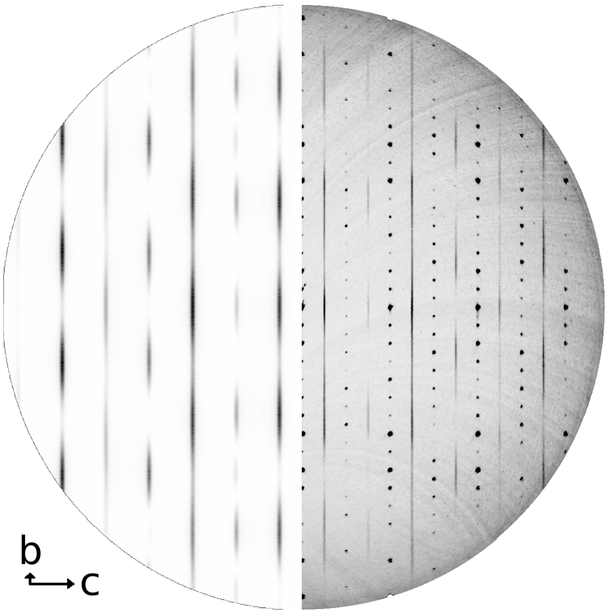

Figure 8: The $(4kl)$ layer: reconstruction from X-ray diffraction data (right, including Bragg reflections). The calculated diffraction pattern (model A) is shown on the left; Bragg intensities are subtracted.
